# Now is it time to implement spacers in cervical cancer brachytherapy?

**DOI:** 10.1093/jrr/rrac031

**Published:** 2022-06-20

**Authors:** Naoya Murakami, Kae Okuma, Tomoyasu Kato, Hiroshi Igaki

**Affiliations:** Department of Radiation Oncology, National Cancer Center Hospital, Tokyo, 104-0045, Japan; Department of Radiation Oncology, National Cancer Center Hospital, Tokyo, 104-0045, Japan; Department of Gynecologic Oncology, National Cancer Center Hospital, Tokyo, 104-0045, Japan; Department of Radiation Oncology, National Cancer Center Hospital, Tokyo, 104-0045, Japan

**Keywords:** uterine cervical cancer, image-guided adaptive brachytherapy (IGABT), spacer

## Abstract

Although the international study on MRI-guided brachytherapy in cervical cancer (EMBRACE-I) demonstrated excellent local control regardless of the T stage, up to 14.6% of grade 3–5 late radiation-related toxicities were observed, which is unacceptable. While the efficacy of hydrogel spacers has been established in prostate radiotherapy, its implementation speed in cervical cancer brachytherapy is relatively slow, despite the fact that several articles have reported its efficacy in cervical cancer brachytherapy. The authors believe that using a spacer in cervical cancer brachytherapy and brachytherapy for other gynecologic malignancies will reduce late radiation-related toxicity and improve patients’ quality of life; therefore, its rapid implementation is required.

Since the introduction of image-guided adaptive brachytherapy (IGABT) for uterine cervical cancer in 2005 [1], the Gynecological (GYN) The Groupe Europen de Curiethrapie and the European Society for Radiotherapy & Oncology (GEC-ESTRO) have continued to investigate the efficacy of IGABT, and in 2021, long-term results of the international study on MRI-guided brachytherapy in cervical cancer (EMBRACE-I) was published in *Lancet Oncology*, demonstrating outstanding clinical results of >90% 5-year local control regardless of T stage [2].

According to the GEC-ESTRO [1, 3], high-risk clinical target volume (CTV_HR_) D_90%_ (minimal dose to 90% of the CTV_HR_) should receive 85 Gy EQD_2_ (the equivalent effective dose in 2 Gy per fraction). While the EMBRACE-I study demonstrated excellent local control, up to 14.6% of grade 3–5 late radiation-related toxicities were observed across all cohorts, going up to 18.4% when only Stage III–IVA toxicities were considered. As a matter of reality, the Japanese guidelines recommendation still does not include 85 Gy EQD_2_ as a prescription goal [4]. Even radical hysterectomy followed by concurrent chemoradiation is unlikely to result in such a high rate of late treatment-related toxicities [5]. It has been clearly demonstrated that if >85 Gy EQD_2_ is delivered to the CTV_HR_, while rectum D_2cc_ < 65 Gy EQD_2_ and bladder D_2cc_ < 80 Gy EQD_2_ are satisfied, excellent local control with minimal late normal tissue toxicities are expected [2, 6, 7]. However, for a certain group of patients, it is impossible to satisfy the above-mentioned dose constraints while delivering >85 Gy EQD_2_ to the CTV_HR_, and for such patients no solution is currently provided in the guidelines [1, 3, 8, 9]. Interstitial brachytherapy or the hybrid of intracavitary and interstitial brachytherapy (HBT) would offer help for meeting such strict dose constraints, particularly in the case of large or irregularly shaped tumors [10–12]. The Japanese guidelines recommend using a central shield (CS) following 20–40 Gy of whole pelvic radiation therapy to avoid extremely high radiation exposure for the rectum and bladder [4]. As a result, it is not difficult to satisfy dose constraints recommended for the rectum and bladder in the Japanese dose schedule. In calculating the total dose of external beam radiation therapy (EBRT) and brachytherapy, the dose contribution of CS is generally ignored, although the dose contribution of CS to CTV is ineligible and it has been shown that 5–10% of the CS dose is actually delivered to the CTV [13, 14]. Even so, however, to achieve CTV_HR_ D_90_ > 85 Gy, either the CS dose should be reduced or the brachytherapy dose should be increased than recommended in the Japanese guidelines [4].

Because 25% of the patients in the EMBRACE-I study received <85 Gy EQD_2_ to the CTVHR D_90%_, the ongoing EMBRACE-II study requires 85 Gy EQD_2_ to the CTV_HR_ D_90%_ to be more strictly observed for further improvement [6]. Although EBRT must be delivered in the form of intensity-modulated radiation therapy (IMRT) or volumetric modulated arc therapy (VMAT) in EMBRACE-II, the anterior wall of the rectum or the bladder base would inevitably receive a full dose of EBRT because a margin must be added to account for organ motion or set-up, error even with IMRT or VMAT. Therefore, the rate of severe radiation-related rectum or bladder toxicities in the EMBRACE-II study would be expected to be similar to that in EMBRACE-I.

It has been reported that spacers have a favorable efficacy in the management of prostate radiotherapy in both EBRT and brachytherapy [15–17]. Although usage of spacers in cervical cancer brachytherapy has already been reported [18–22], it must be noted that its implementation speed is quite slow when compared to prostate radiotherapy. Even though adverse events involving hydrogel spacers in prostate radiotherapy are uncommon, they have been reported [23, 24]. This could be because the approved prostate hydrogel is composed of polyethylene glycol and is constructed in such a way that it will remain in place for several months until total prostate radiation therapy is completed. As a result, if it is inserted in the wrong space, it will cause unexpected adverse events because a long time is required to dissolve the material in the tissue. In contrast, the hydrogel spacer which our group has been using is made of hyaluronate acid and is rapidly absorbed over several days. Therefore, even if it is inserted incorrectly, it will be absorbed quickly and will not cause any significant tissue damage. While the female and male pelvises are anatomically different, the vagina is much softer and thinner than the prostate, and a much larger spacer exists anterior to the rectum in the female pelvis than in the male pelvis. Therefore, it is much easier to insert a hydrogel spacer between the rectum and the vagina than in the male pelvis. Our group has been using a hyaluronate gel product that is already approved for the treatment of knee osteoarthritis.

It is true that spacer gel injection needs to be guided by trans-rectal ultrasound (TRUS) to guide a needle to the right anatomic position. However, it is presumed that the majority of brachytherapy operation rooms do not have a TRUS dedicated to brachytherapy. Thanks to recent lobbying activities to increase medical remuneration points regarding image-guided brachytherapy, assuming that a gynecologic patient requires four brachytherapy treatments, one patient receiving intracavitary brachytherapy (ICBT) or HBT would cost 803 000 and 1 513 000 yen, respectively, including management, radioisotope source and irradiation costs. Even after subtracting the annual mandatory expenses such as radioisotope costs incurred every three months and maintenance and inspection costs, if more than 11 patients with ICBT or five patients with HBT are treated within a year, a hospital will have an annual surplus of over five million yen, which will enable the hospital to purchase a new TRUS machine for brachytherapy.

To obtain an additional indication for the Japanese universal health insurance to cover the cost of hydrogel spacer usage for female pelvic brachytherapy, it is always the case that a prospective clinical trial is required to demonstrate the efficacy of the hydrogel spacer. The authors reasoned that because conducting such a clinical trial is time-consuming and expensive, and its efficacy is readily apparent by creating a physical space between the high-dose area and the organs at risk, when we publish articles demonstrating the efficacy of hydrogel spacer, the adoption of hydrogel spacer in female pelvic brachytherapy will be accelerated. However, contrary to our expectations, the hydrogel spacer has not been used in female pelvic brachytherapy other than at our hospital. Additionally, the pharmaceutical company is unwilling to obtain an additional indication for the Japanese universal health insurance coverage, owing to the fact that the market for brachytherapy is much smaller than the market for osteoarthritis and the drug is extremely inexpensive. As a result, the authors now realize that even if it takes a long time to perform a physician-led prospective clinical trial, it will be a faster way to accelerate the implementation speed of hydrogel spacer usage in female pelvic brachytherapy.

Nevertheless, the authors believe that using a gel spacer to safely increase the tumor dose while sparing doses to the organs at risk is critical, and that its rapid implementation in cervical cancer and other gynecological brachytherapy is an urgently needed.
